# Integration of the metabolome and transcriptome reveals the molecular mechanism of drought tolerance in *Plumeria rubra*

**DOI:** 10.3389/fgene.2023.1274732

**Published:** 2023-09-18

**Authors:** Rong Sun, Shan Liu, Jinglei Gao, Lihua Zhao

**Affiliations:** Department of Biological Engineering, College of Biological and Chemical Engineering, Panzhihua University, Panzhihua, China

**Keywords:** drought tolerance, Plumeria rubra, hormone, organic acids, flavonoids

## Abstract

*Plumeria rubra* L. cv. Acutifolia is an ornamental tree that displays a good drought-tolerance level. However, the molecular mechanisms of *P. rubra* adaptation to drought stress remains unclear. Here, drought-simulating pot experiments were conducted to explore drought stress response mechanism of *P. rubra*. Transcriptome analysis revealed 10,967 differentially expressed genes (DEGs), 6,498 of which were increased and 4,469 decreased. Gene Ontology (GO) analysis revealed that the DEGs were enriched in binding category, in metabolic process category, and in catalytic activities category. The Kyoto Encyclopedia of Genes and Genomes (KEGG) analysis showed that 9 pathways were significantly enriched, including biosynthesis of secondary metabolites (ko01110), plant hormone signal transduction (ko04075) and so on. In addition, the transcription factor families of *AP2/ERFs*, *bZIP*, and *C2H2* were significantly upregulated while the families of *bHLH*, *MYB-related,* and *NAC* were significantly downregulated. Moreover, the results of metabolomics analysis indicated that some compounds were accumulated under drought stress, especially flavonoids. Overall, it was speculated that under drought stress, *P. rubra* first activates the plant hormone signal transduction pathway to regulate hormone contents. Then osmotic regulating substances such as organic acids and amino acids are accumulated to maintain osmotic balance. Finally, flavonoid levels are increased to scavenge reactive oxygen species. These results preliminarily revealed the molecular mechanisms adopted by *P. rubra* in response to drought stress.

## Introduction

Drought due to global warming, water shortages, and soil desertification has become one of the main abiotic limiting factors that limits plant survival and growth (Fathi and Tari, 2016; Zandalinas et al., 2018). Drought leads to plant height shortening, node number reduction, leaf area reduction, dry matter accumulation reduction, growth period extension, and yield reduction (Guo et al., 2016; Maatallah et al., 2016; Okunlola et al., 2017). Currently, more than 50 countries are suffering from drought stress (Wang, 2011). Therefore, protecting and excavating drought-resistant germplasm resources and improving drought resistance in plant breeding are important goals (Hu and Xiong, 2014).

In order to respond to drought stress plants had evolved a series of complex mechanisms. These include modulated osmoregulatory metabolites contents, accumulated abscisic acid, increased activities of antioxidant enzymes, and induced expression of drought stress-related genes (Oono et al., 2003; Verslues et al., 2006; Goswami et al., 2013; Osakabe et al., 2014). To data, considerable research on the physiological and biochemical mechanisms of plant drought resistance has been reported. However, there is fewer research on molecular mechanism of plant drought resistance (Wei et al., 2020). In order to better breed drought-resistant plant varieties, the molecular mechanism is necessary to clarify.

As technological innovations, transcriptome analysis and metabolome analysis are powerful strategies for exploring the genes and secondary metabolites involved in the environmental stress response. For example, combined analysis of transcriptomics and metabolomics data was used to study the *Carthamus tinctorius* drought tolerance. The result indicated that fourteen candidate genes, three metabolites, and eight metabolic pathways maybe correlated with *C*. *tinctorius* drought-tolerance (Wei et al., 2020). Li et al. (2021) revealed the ko04075 (plant hormone signal transduction) pathway and ko00940 (phenylpropanoid biosynthesis) pathway associated with *Cucurbita maxima* cold tolerance by integrating transcriptome and metabolome analyses. A transcript and metabolite profiling approach was used to investigate the candidate genes and metabolites involved in grapevine berries respond to drought. Savoi et al. (2016) reveals that white grapes respond to drought by stimulating the production of phenylpropanoids, of volatile organic compounds and of carotenoid zeaxanthin. Hence, integration of the transcriptome and metabolome is a powerful strategy for explaining the molecular mechanisms associated with the drought resistance of plants.

*Plumeria rubra* is an ornamental tree species favored for planting in parks and gardens due to its brightly colored and fragrant flowers. It can grow normally in a strong light, high temperature, and shaded environment (Yang et al., 2014). Panzhihua launched a pilot project in the typical dry-hot valley area of Hongge to investigate whether *P. rubra* can be used for the ecological restoration of rocky deserts. The results showed that, after experiencing high temperatures and drought, the *P. rubra* survival rate still reached >90%. In addition, the trees developed root systems, showed a fast growth rate, and excellent soil fixation effective, exhibiting a remarkable ability to address rocky land desertification (Wu, 2019). Thus, *P. rubra* has good drought tolerance. Nevertheless, there have been few studies on this species’ responses to drought stress. Here, transcriptomics and metabolomics profiles of *P. rubra* leaves for a drought stress treatment and a control treatment were established to screen the differentially expressed genes (DEGs) and differential metabolites (DMs). Then, the transcriptomics and metabolomics data were integrated in order to elucidate the molecular mechanisms mediating the *P. rubra* drought stress responses. The results provided useful information and potential scientific value for understanding how plants adapt their growth under drought conditions.

## Materials and methods

### Plant materials and experimental design

Cuttings of *P. rubra* with similar growth statuses were selected and planted in the nursery of Panzhihua University. Then, the scions were placed at 25°C for cultivation. When the scions were 3 months old, they were selected as study materials for further experiments. The drought-simulating pot experiments were then conducted. Before drought treatment, water was poured thoroughly into the soil of each pot containing the plants, and the materials were then divided into two groups: the control group (TCK, soil volumetric water content 15%–20%) and the drought stress group (TSD, soil volumetric water content <5%). The Field Scout TDR 150 soil moisture meter (Spectrum, United States) was used to determine the volumetric water content in the soil. After about 15 days, when reaching the level of the TSD group, the tender leaves were selected for transcriptomics analysis and the mature leaves were selected for metabolomics analysis. Six scions in the same treatment were deemed to be one experimental unit. The experiments were replicated three times.

### Metabolome and transcriptome profiling

After the samples were collected, they were quickly frozen in liquid nitrogen and sent to the Wuhan Metware Biotechnology Co., Ltd. (Wuhan, China) on dry ice for transcriptome and metabolome profiling. For transcriptome analyses, after extracting of total RNA, first the RNA purity was determined by a NanoPhotometer N80 (Implen, Germany), and the integrity of the RNA was analyzed using an Agilent 2,100 Bioanalyzer (Agilent, United States). Then, random hexamers were used to synthesize first-strand cDNA, and double-stranded cDNA were purified by AMPure XP Beads (Beckman Coulter, United States). Finally, an Illumina sequencing HiSeqTM 2,500 platform was used for library sequencing.

For metabolome profiling, first freeze-dried the samples and crushed them. Then, 70% methanol was used to extract the samples at 4°C overnight. Finally, centrifugated and filtrated the extracts for ultra-performance liquid chromatography tandem mass spectrometry (UPLC-MS/MS) analyses. UPLC-MS/MS analyses were performed using a SHIMADZU Nexera X2 (Shimadzu, Japan) system coupled with an Applied Biosystems 4,500 QTRAP (Thermo Fisher Scientific, United States).

### Identification and annotation of DEGs for transcriptome analysis

The fragments per kilobase of transcript sequence per million mapped reads (FPKM) were calculated to assess gene expression levels. The distances between the samples was calculated by principal component analysis (PCA). Pearson’s correlation coefficient (PCC) was used to evaluate the linear association between the gene expression levels. The DEseq2 package was used to calculate each transcript expression level (Anders and Huber, 2010). In two samples, the genes with a false discovery rate (FDR) of <0.05 and |log_2_ FC| ≧ 1 were defined as DEGs. The DEGs were then determined by Gene Ontology (GO) analysis and the Kyoto Encyclopedia of Genes and Genomes (KEGG) pathway enrichment analysis. The KEGG category enrichment with a *p*-value of ≤0.05 was considered to be significantly enriched. The iTAK package was used to identify transcription factors (TFs).

### Identification of DMs

Analyst 1.6.3 software was used to processe the mass spectrometric data. The quality control samples were combined by equal volumes of each experimental sample. PCA and partial least squares-discriminant analysis (PLS-DA) were used to screen DMs. The metabolites with a variable importance in projection (VIP) value ≧ 1 and |log_2_ FC|≧ 1 were considered to be DMs. Then, KEGG compound database was used to annotate the DMs and mapped the DMs to KEGG pathway database.

### Integrative analysis of the metabolome and transcriptome

PCCs between related genes and metabolites were calculated to construct the regulatory network. Canonical correlation analysis (CCA) was perform between DEGs and DMs in each pathway.

## Results

### Transcriptomic analysis of *P. rubra* under drought stress

**FIGURE 1:**
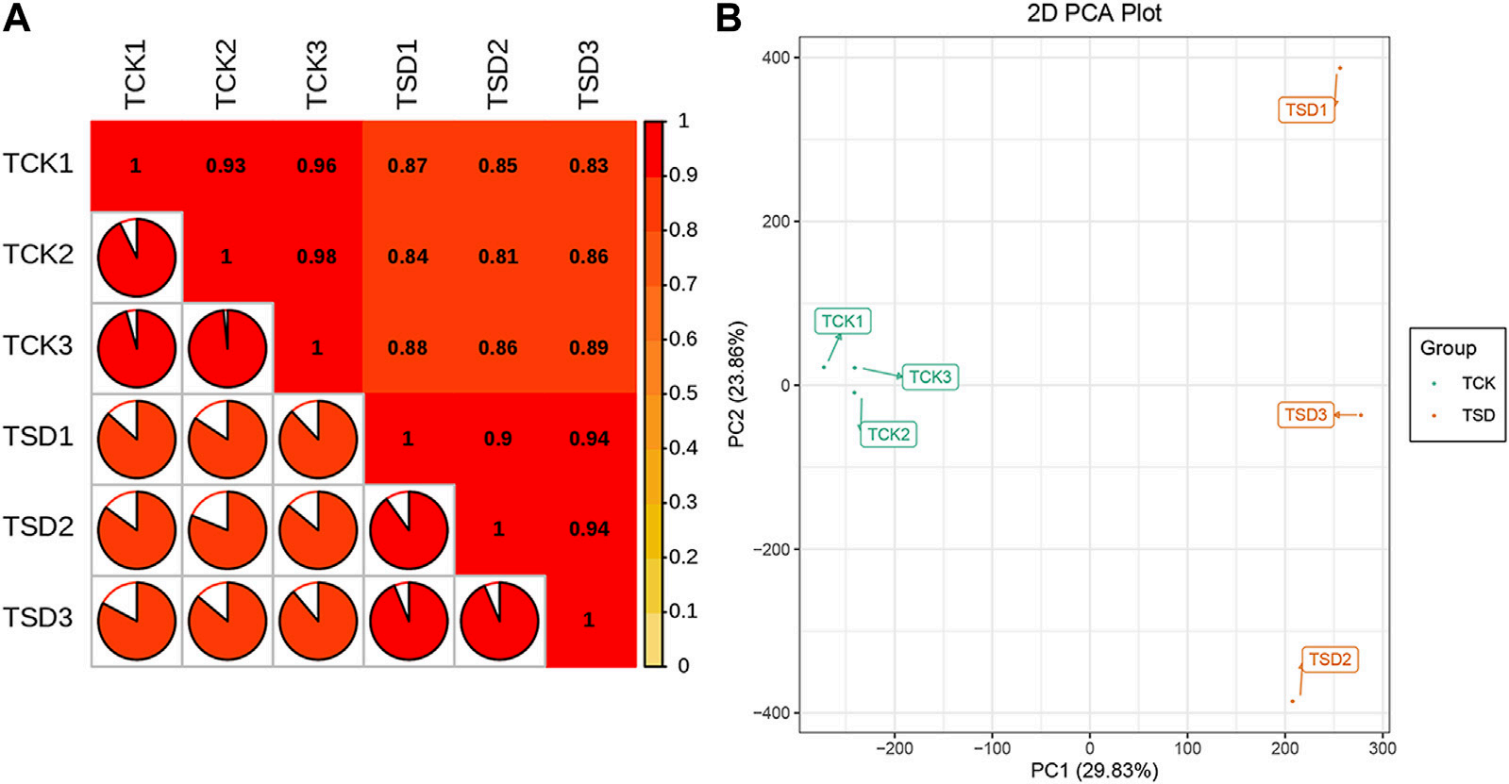
Correlation statistics **(A)** and principle component analysis **(B)** between samples in the control group (TCK) and the drought stress group (TSD).

To investigate the discrepancies in the *P. rubra* leaves under drought stress, two treatments of *P. rubra* with three replicates were selected for transcriptomic analysis on an Illumina HiSeqTM 2,500 platform. The high-quality bases from six samples were 59.59 Gb in total. And the clean data of each sample was at least 9.25 Gb (Table 1). The Q30 ratio in all samples were >93%, and the GC content was >43%. The PCCs among each replicate were all >0.9 (Figure 1A), which indicated that the replicates has satisfactory repeatability and accuracy. PCA results indicated that samples between the two treatments were clearly separated and that there was significant difference in transcripts between the two treatments (Figure 1B). Overall, these results indicated that the data were suitable for the subsequent analysis.

**TABLE 1:** Statistics of the transcriptome data of *Plumeria rubra*.

Sample	Clean reads (M)	Clean base (G)	Error rate (%)	Q30 (%)	GC (%)
TCK1	66,720,564	10.01	0.03	94.19	43.07
TCK2	71,658,544	10.75	0.03	93.96	43.15
TCK3	69,144,440	10.37	0.03	93.98	43.15
TSD1	61,640,834	9.25	0.03	94.15	43.01
TSD2	65,133,110	9.77	0.03	94.22	43.05
TSD3	62,922,084	9.44	0.02	94.61	43.24

### Identification of DEGs in response to drought stress

**FIGURE 2:**
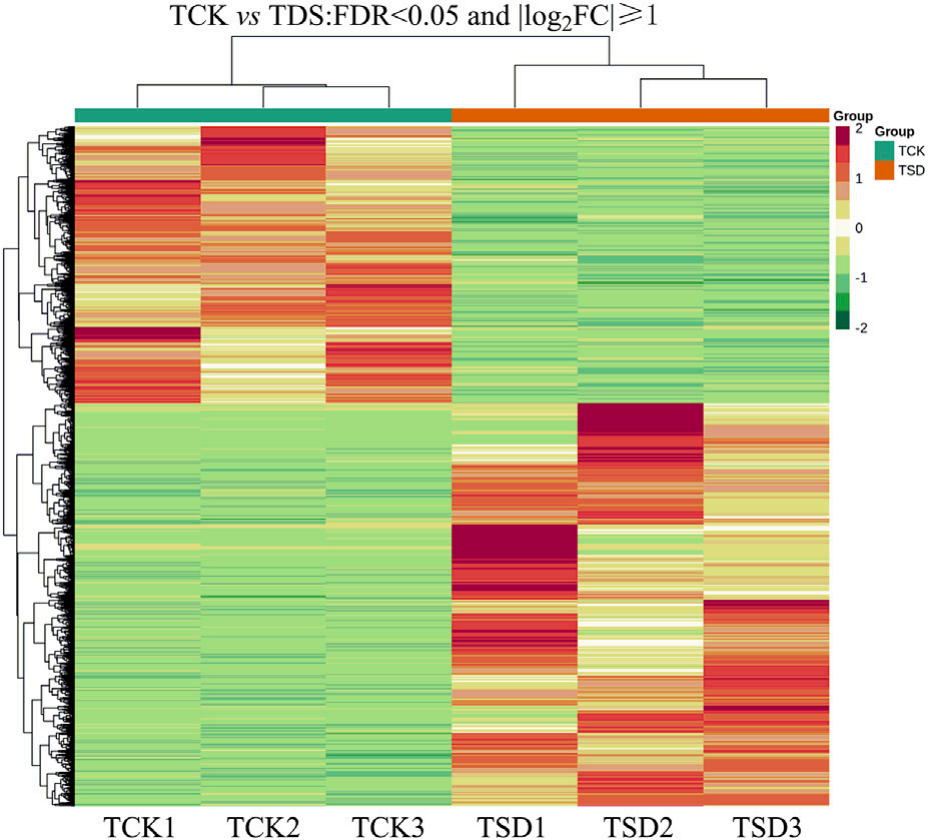
Heat map of the TCK vs*.* TSD (false discovery rate <0.05, |log_2_ FC| ≧1). The red color in the heat map indicates upregulated genes and green indicates downregulated genes.

The DEGs from the two treatments were determined. The reads with |log_2_ FC|≧ 1 and a FDR <0.05 were selected to annotate the DEGs. A total of 10,967 DEGs were detected, with 4,469 downregulated and 6,498 upregulated genes (Figure 2).

**FIGURE 3:**
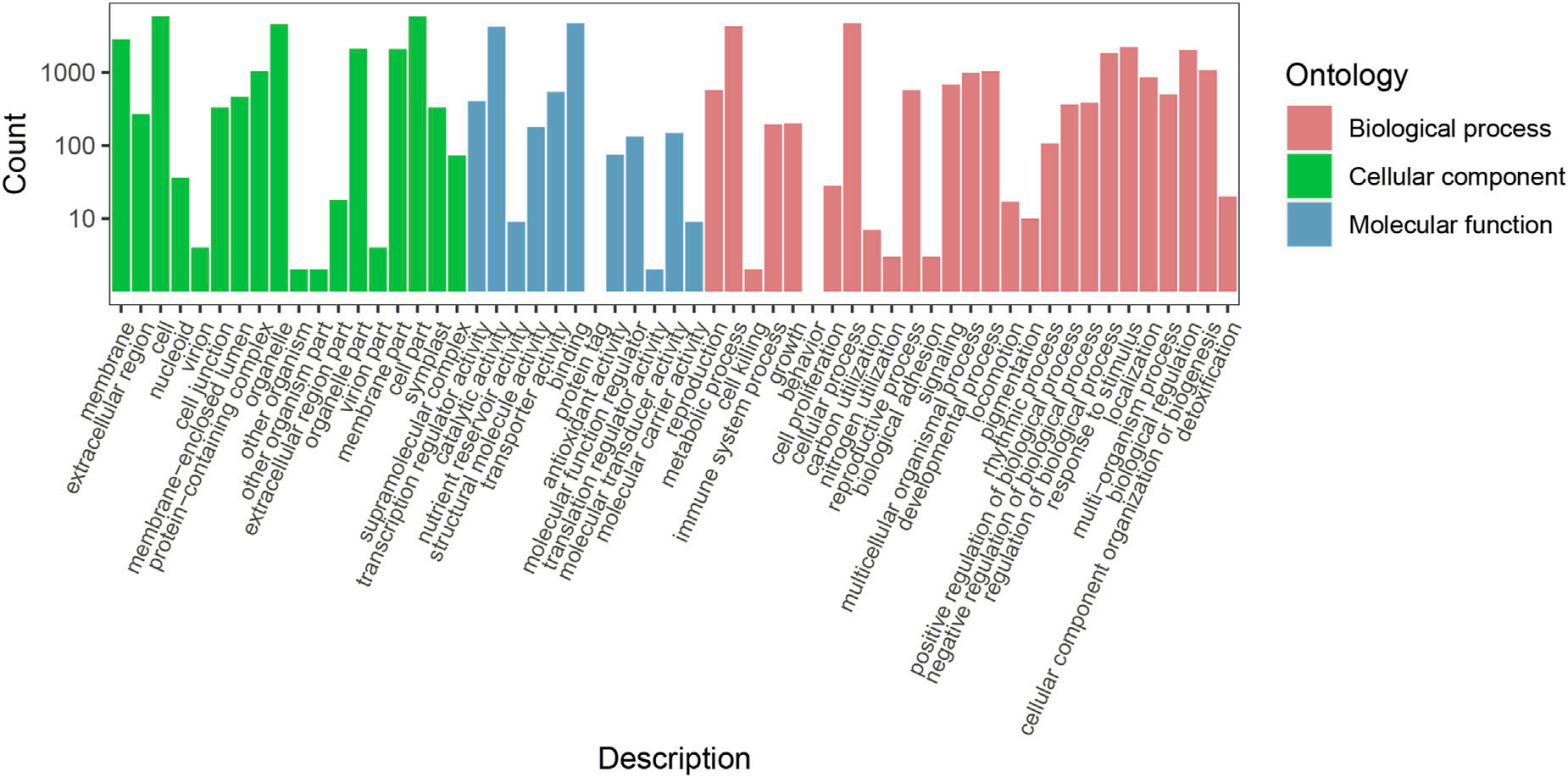
Functional classification of *Plumeria rubra* differentially expressed genes by GO enrichment analysis.

GO enrichment analysis was used to obtain the function information of the DEGs. The results showed that 7,930 DEGs (including 4,760 upregulated and 3,170 downregulated genes) were divided into 57 functional groups (Figure 3). A total of 22,739 genes were categorized in the biological process category, 25,829 genes in the cellular component category, and 10,446 genes in the molecular function category. In the biological process category, 4,705 (20.69%), 4,274 (18.79%), 2,234 (9.82%), and 2,035 (8.94%) genes were found to be involved in cellular process (GO: 0.009,987), metabolic process (GO: 0.008,152), response to stimulus (GO:0.050,896), and biological regulation (GO:0.065,007), respectively. In the cellular component category, 5,824 (22.54%), 5,812 (22.50%), 4,560 (17.65%), and 2,862 (11.08%) genes were found to be involved in the cell (GO: 0.005,623), cell part (GO: 0.044,464), organelles (GO:0.043,226), and membrane (GO:0.016,020), respectively. In the molecular function category, binding (GO:0.005,488) and catalytic activity (GO:0.003,824) terms had 4,722 (45.20%) and 4,216 (40.35%) genes respectively. These results indicated that a large number of DEGs are involved in the metabolic process, response to stimulus, biological regulation, and catalytic activities, which was conducive to the subsequent screening of functional genes in this study.

**FIGURE 4:**
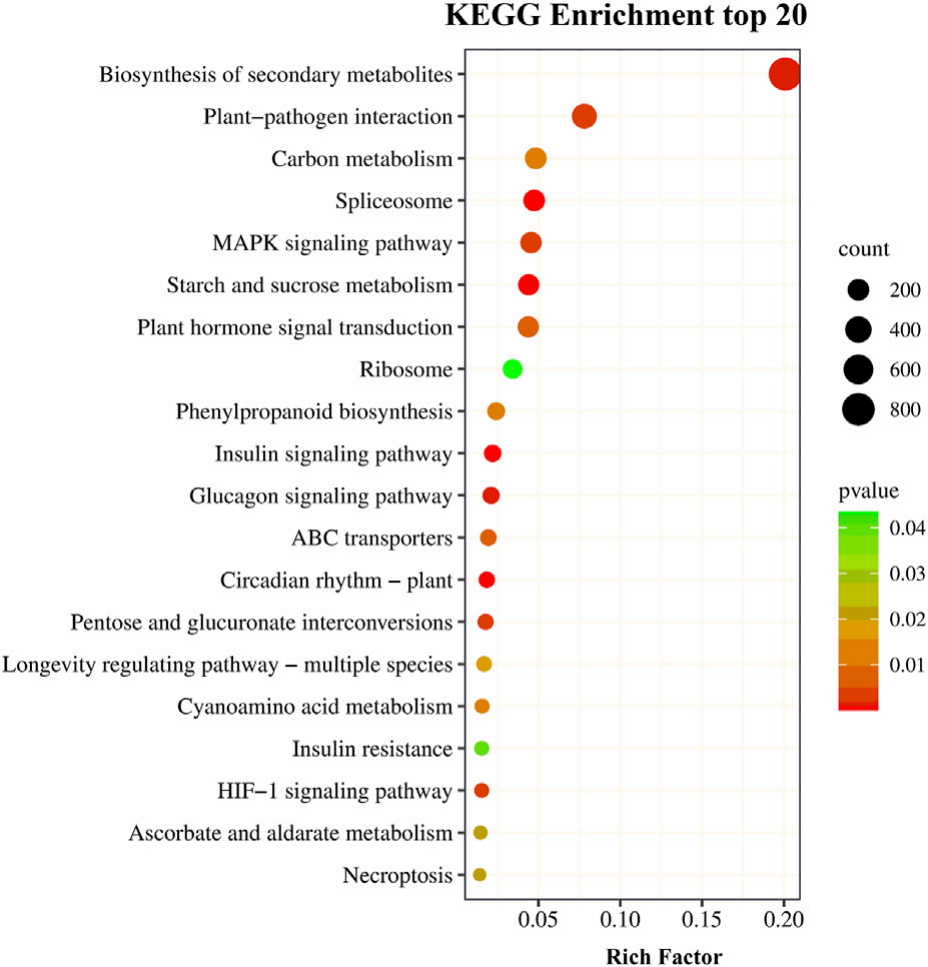
The top 20 KEGG pathways for drought responsive differentially expressed genes.

The KEGG is a integrative database, making it an effective tool for elucidating the biological function of DEGs. A total of 4,177 DEGs were annotated using the KEGG database that were involved in 388 metabolic pathways categorized into 6 classifications (Supplementary Figure S1). The most abundant pathways were the metabolic pathways (ko01100) followed by the biosynthesis of secondary metabolites (ko01110). A *p*-value of ≤0.05 indicated the pathways with significantly expressed DEGs. The top 20 significantly enriched pathways (*p* ≤ 0.05) are shown in Figure 4. Among them, ko00500 (starch and sucrose metabolism), ko00940 (phenylpropanoid biosynthesis), ko01110 (biosynthesis of secondary metabolites), ko01200 (carbon metabolism), ko03010 (ribosomes), ko03040 (spliceosomes), ko04016 (MAPK signaling pathway-plant), ko04075 (plant hormone signal transduction) and ko04626 (plant-pathogen interaction) enriched >100 DEGs, suggesting that these pathways may be closely related to drought stress in *P. rubra*.

### TF gene expression changes under drought stress in *P. rubra*

**FIGURE 5:**
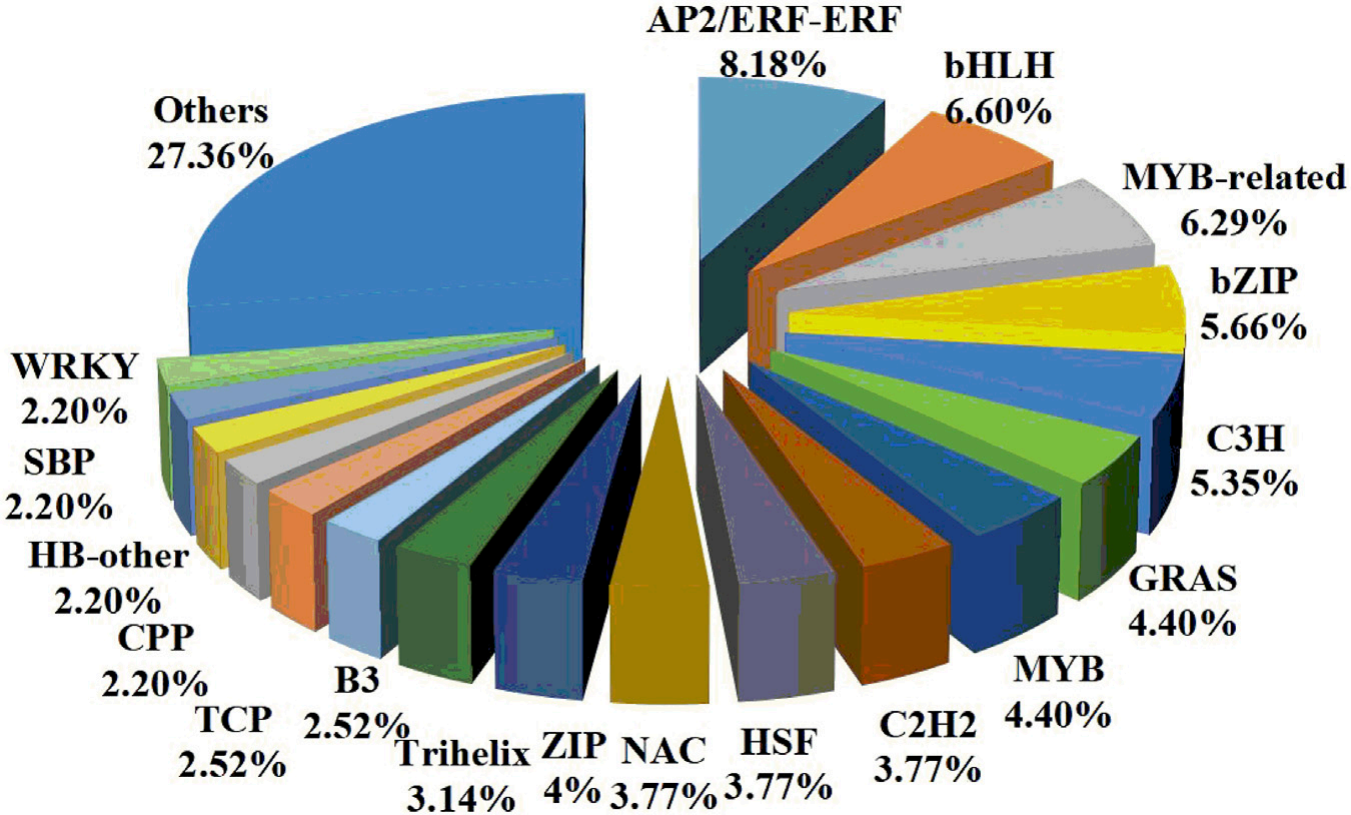
Classification of the drought-responsive transcription factors in *Plumeria rubra.*

TFs are considered to be the most important regulators involved in regulatory mechanisms. Therefore, 318 DEGs (224 upregulated and 94 downregulated) encoding putative TFs were further identified. These TFs were classified into *AP2/ERFs, bHLH, MYB-related, bZIP, C3H, GRAS, MYB, C2H2, HSF, NAC, HB-HD-ZIP, Trihelix*, and other TF families (Figure 5). Among them, 72 TFs were uniquely expressed in the control treatment and 25 TFs were uniquely expressed in the drought stress treatment. The results of the |log_2_ FC| calculation showed that putative *AP2/ERFs, bZIP*, and *C2H2* were significantly upregulated under drought stress. In addition, putative *bHLH*, *MYB-related*, and *NAC* were significantly downregulated under drought stress. Therefore, it was speculated that these TFs might be the drought-tolerant response genes.

### Detection and profile analysis of metabolites under drought stress

**FIGURE 6:**
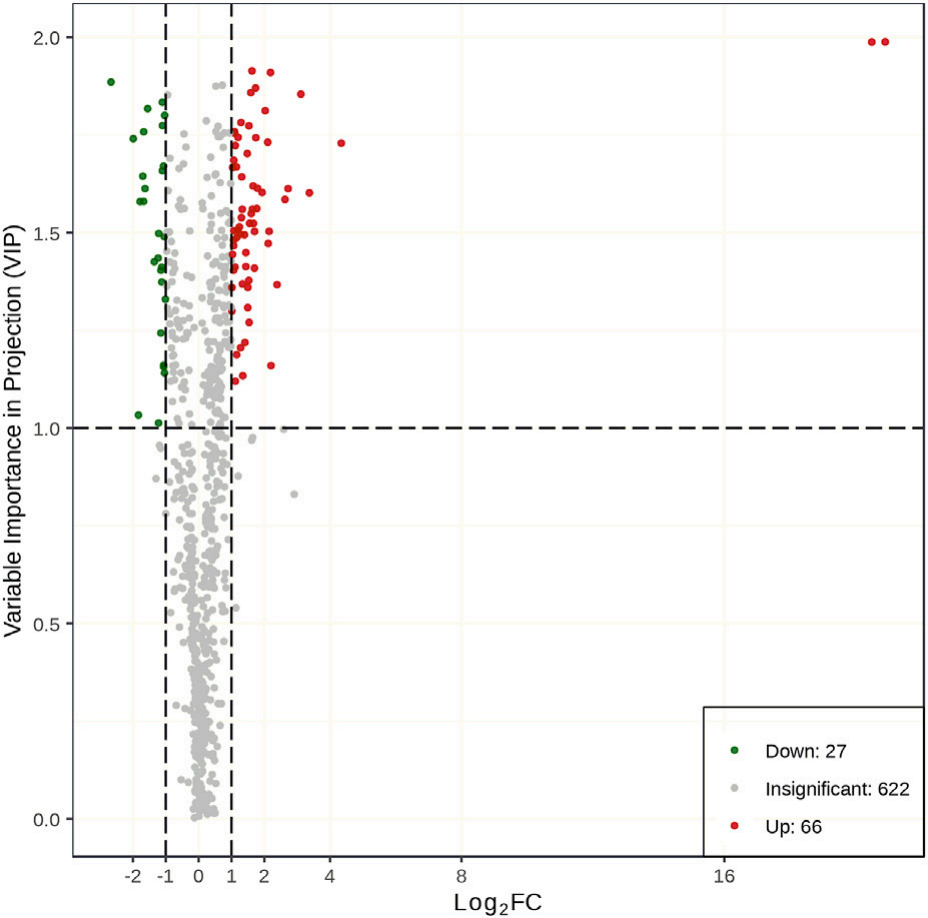
The differential metabolites between the TCKs and TSDs.

**FIGURE 7:**
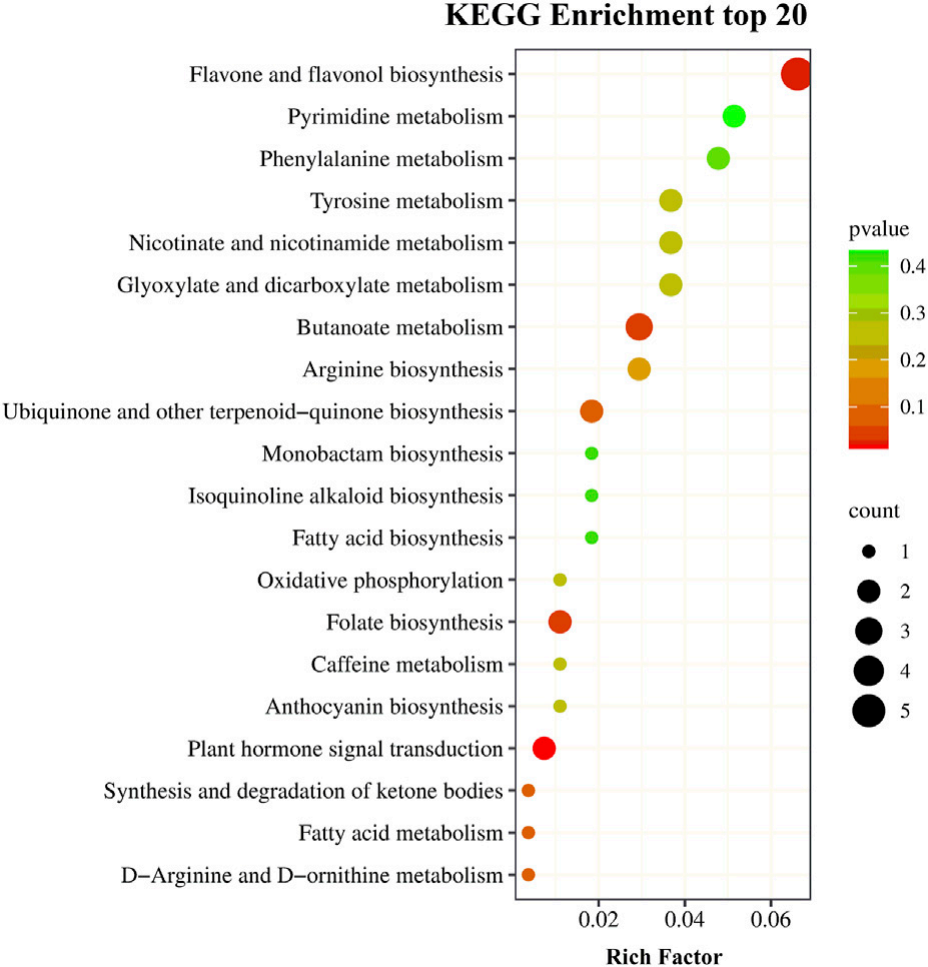
The top 20 KEGG pathways for drought-responsive differential metabolites.

The same samples as those used for transcriptomics analysis were used to perform metabolomics analysis on the UPLC-MS/MS platform. There were 715 metabolites detected. The PCCs among each replicate were all >0.85 (Supplementary Figure S2), which conformed to repeatability requirements. The PLS-DA analysis results indicated that samples were clearly separated between the two treatments, and there were significant inter group differences (Supplementary Figure S3). The metabolites with a VIP value ≧ 1 and |log_2_ FC| ≧ 1 were defined as DMs. A total of 93 metabolites (66 upregulated and 27 downregulated) showed a significant difference between the drought stress and control conditions (Figure 6). Among the metabolites with increased expression, the luteolin-3′-O-glucoside and kaempferol-3-O-glucoside (astragalin) in the TSD group displayed a 1,948,037 and 1,467,800 FC compared to the TCK group, respectively. The results of KEGG enrichment analysis showed that 67.86% DMs were annotated in the metabolic pathways (ko01100) and 42.86% DMs were annotated in biosynthesis of secondary metabolites (ko01110). Moreover, plant hormone signal transduction (ko04075), flavone and flavonol biosynthesis (ko00944), folate biosynthesis (ko00790), and butanoate metabolism (ko00650) pathways were the most significantly enriched pathways (Figure 7).

### Integrative analysis of the transcriptome and metabolome

**FIGURE 8:**
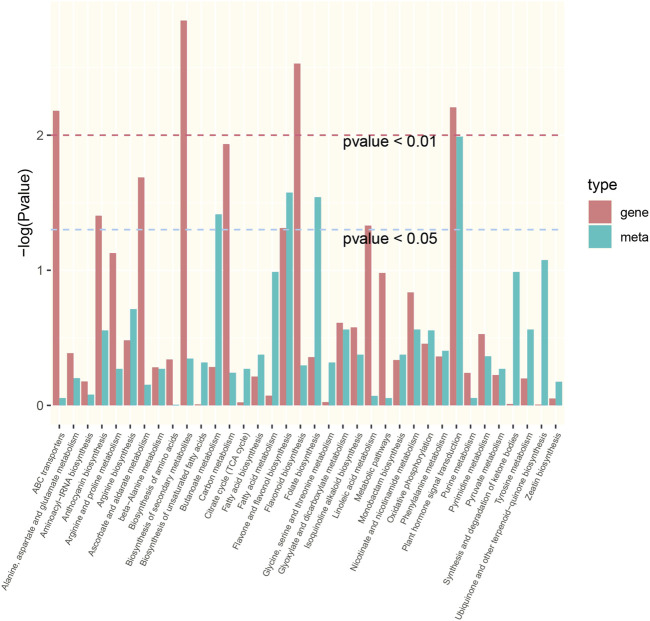
The significantly enriched pathways with either transcriptome or metabolome data with *p* < 0.05.

Integrative analysis of the transcriptome and metabolome was used to accurately screen the DEGs and DMs involved in the response to drought stress in *P. rubra*. A total of 36 significantly enriched pathways were identified (transcriptome or metabolome data with *p* < 0.05) (Figure 8). Among them, the plant hormone signal transduction (ko04075) pathway and flavone and flavonol biosynthesis (ko00944) pathway are significantly enriched both DEGs and DMs. Furthermore, the interaction networks between DEGS and DMs were constructed according to PCCs. The results showed that 23 pathways had an *R* ^2^ value of >0.8. Finally, canonical correlation analysis was performed on the DEGs and DMs, indicating that six of the 23 pathways had canonical correlation, including plant hormone signal transduction (ko04075), glyoxylate and dicarboxylate metabolism (ko00630), arginine biosynthesis (ko00220), flavonoid biosynthesis (ko00941), tyrosine metabolism (ko00350), and phenylalanine metabolism (ko00360).

In the plant hormone signal transduction pathway, 183 and 2 correlated DEGs/DMs were obtained. By comparing TSD with the TCK groups, it was found that the cytokinin (CK) content increased by 2.51-fold while the salicylic acid (SA) content decreased. In the glyoxylate and dicarboxylate metabolism pathway, 61 and 2 correlated DEGs/DMs were obtained, with hydroxypyruvic acid and tartronate semialdehyde contents both increasing under drought stress. In the arginine biosynthesis pathway, 33 and 2 correlated DEGs/DMs were obtained, and the changes in arginine contents were 2.54-fold. In the tyrosine metabolism pathway, 26 and 2 correlated DEGs/DMs were obtained. In the flavonoid biosynthesis and phenylalanine metabolism pathway, 31/2 and 24/2 correlated DEGs/DMs were obtained, respectively. Moreover, the contents of maleic acid, fumaric acid, neohesperidin, and epigallocatechin accumulated in the TSD group.

Therefore, it was speculated that under drought stress, *P. rubra* first activates the plant hormone signal transduction pathway to regulate the hormone contents. Then, organic acids and amino acids involved in osmoregulation accumulate to maintain the osmotic balance. Finally, the flavonoid contents increase to scavenge reactive oxygen species (ROS).

## Discussion

Plants’ responses to drought stress are complex, and are related to a large number of genes and metabolites changes (Tardieu and Tuberosa, 2010). To our knowledge, it is the first time that study drought resistance in *P. rubra* by combining of transcriptomics and metabolomics analysis. The results indicated that there has significant difference in gene expression and metabolite profiles between two treatments, especially in the genes and metabolites involved in the plant hormone signal transduction, glyoxylate and dicarboxylate metabolism, arginine biosynthesis, flavonoid biosynthesis, tyrosine metabolism, and phenylalanine metabolism pathways.

### Plant hormone signaling involved in drought stress

Plant hormones play significant roles in response to various stresses, particularly abscisic acid (ABA) (Verma et al., 2016). ABA is usually considered as the primary stress-induced hormone. However, increasing evidence suggests that other hormones are also involved in response to stresses, such as AUX, CK, MeJA, BR, and SA (Klessig et al., 1993; Pospíšilová et al., 2000; Pauwels et al., 2008; Jiang et al., 2013; Rahman, 2013). In this study, the genes in the CK signaling pathway were all upregulated, which resulted in CK accumulation. The results are consistent with those of recent studies showing that CK plays an important role in the drought response. Wang H. L. et al. (2022) used a senescence and drought-inducible promoter to drive key CK synthesis genes, which induced the synthesis of CKs, leading to enhanced drought tolerance. Similarly, Wang N. et al. (2022) investigated the CK function in wheat drought tolerance by characterizing the isopentenyltransferase (*IPT*) genes in CK biosynthesis. The results indicated that *TaIPT8* mutants and drought-induced overexpression plants experienced the beneficial effects of CKs for wheat drought tolerance. Furthermore, in this study, the SA contents were downregulated. Considering Wang (2018) study results, here, it is speculated that when *P. rubra* is exposed to drought stress, the free SA may change into a binding state to reduce toxicity.

### Organic acids and amino acids in response to drought stress

Previous studies have shown that organic acids, especially succinic acid, malic acid, and galacturonic acids, enhance the plant drought tolerance (Khan et al., 2020). For example, malic acid can improve the drought tolerance of *Ziziphus jujuba* (Gao et al., 2012). Galacturonic acids play an important role in oxidative damage under drought stress. Succinic acid have a vital role in plants in response to various stresses and enhances tolerance (Yue et al., 2018). Amino acids can act as regulatory and signaling molecules in plant reactions to many stresses (Alessandro and Alessandro, 2006). In the present study, under drought stress, the level of organic acids and amino acids elevated by 2.02-2.89-fold. According to the above, the accumulation of these amino acids and organic acids is very important for *P. rubra* drought tolerance.

### Flavonoids in response to drought stress

Drought stress interferes with the dynamic redox balance of plant cells, resulting in the excess production of ROS, which can lead to damage to lipids, proteins, and nucleic acids, and even cell death (Mittler, 2002; Del Rio, 2015). Flavonoids are natural antioxidants that can help to remove ROS to improve drought tolerance (Agati et al., 2012). Moreover, other studies have reported that the exogenous application of phenylalanine can activate ROS-scavenging systems to promote chilling tolerance in tomato plants (Aghdam et al., 2019). Here, the contents of neohesperidin and epigallocatechin significantly changed, indicating that ko00941 and ko00360 are stress-resistant pathways. Flavonoids play an important role in the drought resistance of *P. rubra*.

## Conclusion

In order to illuminate the response mechanisms of *P. rubra* under drought stress, integrative analysis of the transcriptome and metabolome was carried out. The comparative analysis showed a total of 10,967 DEGs, 318 TFs, and 93 DMs were identified. Moreover, drought stress broadly activated the pathways for plant hormone signal transduction, glyoxylate and dicarboxylate metabolism, arginine biosynthesis, flavonoid biosynthesis, tyrosine metabolism, and phenylalanine metabolism. These results could help to illustrate the molecular mechanisms in the responses exerted by *P. rubra* to drought stress.

## Data Availability

The original contributions presented in the study are publicly available. This data can be found here: https://www.ncbi.nlm.nih.gov/sra, accession numbers SRR24189298 - SRR24189303. https://www.ebi.ac.uk/metabolights, accession number MTBLS8427.

## Supplementary material

SUPPLEMENTARY FIGURE S1 Functional classification of *Plumeria rubra* differentially expressed genes by KEGG annotated.

SUPPLEMENTARY FIGURE S2 Heat map of PCC between samples.

SUPPLEMENTARY FIGURE S3 PLS-DA score **(A)** and model validation **(B)** of the two treatments.
