# Supplementary figures and images for: Integration of the metabolome and transcriptome reveals the molecular mechanism of drought tolerance in Plumeria rubra
Source: Front Genet. 2023 Sep 18;14:1274732. doi: 10.3389/fgene.2023.1274732 (PMC10544913; doi:10.3389/fgene.2023.1274732)

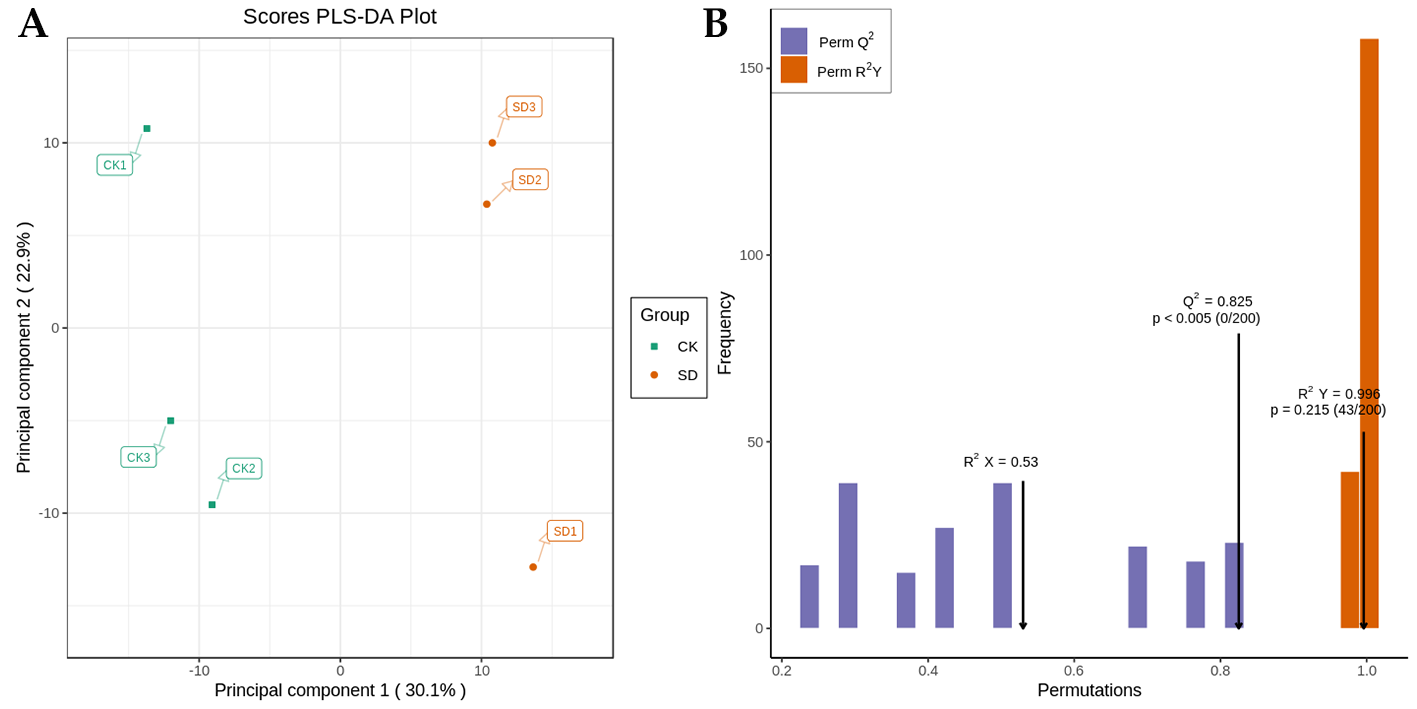

Supplement: Supplementary file 1 [file Image3.TIF]
